# Secondary Prevention via Case Managers in Stroke Patients: A Cost-Effectiveness Analysis of Claims Data from German Statutory Health Insurance Providers

**DOI:** 10.3390/healthcare12111157

**Published:** 2024-06-06

**Authors:** Juliane A. Duevel, Sebastian Gruhn, John Grosser, Svenja Elkenkamp, Wolfgang Greiner

**Affiliations:** AG 5—Health Economy and Healthcare Management, Faculty of Public Health, Bielefeld University, 33615 Bielefeld, Germany

**Keywords:** stroke, transient ischemic attack, TIA, cost-effectiveness analysis, ICER, resource use, claims data

## Abstract

Strokes remain a leading cause of death and disability worldwide. The STROKE OWL study evaluated a novel case management approach for patients with stroke (modified Rankin Scale 0–4) or transient ischemic attack (TIA) who received support across healthcare settings and secondary prevention training from case managers for one year. The primary aim of this quasi-experimental study was a reduction in stroke recurrence. Here, we report the results of a health economic analysis of the STROKE OWL study, conducted in accordance with CHEERS guidelines. The calculations were based on claims data of cooperating statutory health insurance companies. In addition to a regression analysis for cost comparison, the incremental cost-effectiveness ratio was determined, and a probabilistic sensitivity analysis was carried out. In total, 1167 patients per group were included in the analysis. The intervention group incurred 32.3% higher direct costs (*p* < 0.001) than the control group. With a difference of EUR 1384.78 (95% CI: [1.2384–1.4143], *p* < 0.0001) and a 5.32% increase in hazards for the intervention group (HR = 1.0532, 95% CI: [0.7869–1.4096], *p* = 0.7274) resulting in an ICER of EUR 260.30, we found that the case management intervention dominated in the total stroke population, even for an arbitrarily high willingness to pay. In the TIA subgroup, however, the intervention was cost-effective even for a low willingness to pay. Our results are limited by small samples for both TIA and severe stroke patients and by claims data heterogeneity for some cost components, which had to be excluded from the analysis. Future research should investigate the cost-effectiveness of case management interventions for both severe stroke and TIA populations using appropriate data.

## 1. Introduction

Despite wide-ranging developments and medical innovations in prevention and healthcare, strokes remain one of the most common causes of death worldwide [1,2,3]. Of the patients who survive an initial stroke, one-quarter die within the first year, and more than one-third of the survivors are affected by long-term disabilities and require assistance [4,5]. In Germany, the overall incidence for stroke is 226 per 100,000 and increases substantially with age [6,7]. Complications include secondary hemorrhage, heart disease, circulatory disorders, mental disorders, infections, and (chronic) epileptic attacks. Overall, this leads to a high need for treatment, rehabilitation, and long-term care for those affected [8,9]. The risks of dying or long-term disability increase significantly with each subsequent stroke [10]. In addition to the individual burden of stroke, such as permanent impairment in everyday life, and the national economic impact of premature deaths, chronic consequences and disabilities are associated with high financial and personnel challenges for healthcare systems [11,12,13]. Cardiovascular diseases, and predominantly coronary heart disease and stroke, cause the largest direct costs compared to all other diseases [14,15]. In a European comparison, Germany has the second-highest direct per capita healthcare costs due to stroke and the highest indirect per capita expenditures. These are not related to the country-specific stroke incidence or prevalence [16].

In addition to primary stroke prevention, secondary prevention, improved continuity of care, and long-term assistance for stroke patients are also important [2,16]. Despite the high quality of acute care in hospitals, particularly within stroke units, the challenges in discharge to outpatient settings and home care persist in post-stroke management [17]. Particularly in rural regions, there is a risk that patients will not have access to appropriate aftercare. Various interventions aimed at closing this gap are currently under discussion and being tested [18,19,20,21]. According to the relevant literature, the managed care approach, with its various organizational forms and instruments, seems to be suitable for both cost savings and quality improvement [22,23]. Relevant components of efficient case management, especially in the context of chronic diseases, include the longitudinal coordination of healthcare, prevention, and risk screening and the identification of specific needs [22,24].

Therefore, the STROKE OWL (an acronym for the German project title) study evaluated a novel case management approach for stroke patients. Patients experiencing their first stroke or transient ischemic attack (TIA) received support from specially trained case managers across various healthcare settings for one year post-event. Case managers did not perform treatment on their own but monitored existing healthcare use and ensured that patients’ medical needs were met when issues or gaps in treatment were identified, particularly during the transition back to the home environment after hospitalization or rehabilitation. In addition, they provided secondary prevention training to patients and their relatives regarding individual risk factors and necessary lifestyle changes. The aim of this paper is to present the results of the health economic evaluation of the STROKE OWL study. This includes an analysis of resource use and associated costs, as well as a cost-effectiveness analysis with regard to the primary outcome of the STROKE OWL project: stroke recurrence.

## 2. Materials and Methods

### 2.1. Study Design and Data Collection

For the evaluation, a quasi-experimental study was conducted in three regions of Germany. Patients from the intervention region of East Westphalia-Lippe (Ostwestfalen-Lippe (OWL)) were actively recruited and, after they provided informed consent, enrolled in the study and provided with a case manager each [25,26]. For control patients, two regions in North Rhine-Westphalia (Muensterland and Sauerland) were chosen that were geographically and in terms of healthcare provision similar to the study region East Westphalia Lippe.

The control group, which received usual care, was not actively enrolled in the study. Therefore, only statutory health insurance (SHI) claims data were available for the control group. This group was generated by using matching. Three different matching methods were tested with preliminary data. In addition to propensity score matching and optimal full matching, coarsened exact matching was tested [27,28,29,30]. Taking into account all relevant criteria, optimal full matching with a 1:3 ratio (intervention patients to controls) was identified as the most suitable (details submitted elsewhere). A detailed description of the intervention can be found in Supplementary Information (Supplementary File S1 and Figure S1).

We defined patients as having a first-ever stroke or TIA (ICD-10 codes: G45, I60–I64) if no stroke was encoded in the previous 4 quarters. In addition, inclusion and exclusion criteria were defined (see Table 1).

The use of healthcare services, as well as the resulting costs in the intervention and control groups, were presented descriptively and differentiated for each cost component. The observation period covered the first year after an index stroke. However, the index hospitalization was not included in the cost representation, because it is expected that the case manager cannot influence costs incurred before the beginning of the intervention. A temporary loss of earnings, which is usually classified with other indirect cost components as a productivity loss, was instead categorized as a direct cost in our analysis due to German statutory health insurance policies. Employers are responsible for the initial six weeks of sick leave payments; thereafter, compensation is provided by statutory health insurers to their members. This classification occurs once the employer’s obligation ceases after the six-week period. The evaluation and reporting were conducted in accordance with CHEERS guidelines [32]. The health economic analysis was conducted from a payer’s perspective and was based on the total annual healthcare costs per patient in euros (2022). Healthcare resource use was quantified based on different reference values, differentiated by cost component (see Table 2).

In addition to direct costs, productivity losses (indirect costs) for both control and intervention groups were calculated using SHI claims data, using the friction cost approach [33]. According to this approach, productivity losses for each individual are calculated by multiplying the average compensation of employees in Germany and the fraction of the year spent on sick leave by that individual (=number of individual sick leave days divided by 365). In this calculation, the number of individual sick leave days was capped at 99 days, the average vacancy time in the German labor market [34,35]. The calculations showed a mean value per lost day of EUR 119.59 in 2018, EUR 123.64 in 2019, EUR 124.19 in 2020, and EUR 128.01 in 2021.

### 2.2. Intervention Costs

As the intervention has not yet been implemented in the healthcare system, a hypothetical cost calculation was conducted in collaboration with the SHI companies. The full intervention included mandatory elements (such as enrollment and assessment) and optional modules (like rehabilitation visits), as detailed in Table 3. This cost simulation informed the potential intervention costs for the subsequent cost-effectiveness analysis.

Optional rehabilitation visits were carried out for 63% of the patients in the intervention group (see Table 3). In almost 7% of those affected, the determination of a separate neuropsychological need was necessary. Telephone contacts between the case managers and their patients or relatives were carried out on average 2.3 times during the year of support, whereas (additional) personal contacts were used on average only about 1.2 times. This resulted in mean intervention costs of EUR 552.05 per patient (SD: EUR 117.78).

### 2.3. Statistical Analyses

Mean costs between intervention and control groups were initially analyzed using a test-based approach. Depending on whether the data were normally distributed, *t*-tests or Wilcoxon rank sum tests were performed. Weighting from matching was included in all analyses. A significance level of 0.05 was used for all analyses performed. A generalized linear model (glm), where costs were log-transformed, was applied. This log-linear regression model was fitted with backward selection using the Akaike information criterion (AIC). Bonferroni correction was used to control for multiple testing. The fit of the model was tested with residual analysis. All predictors that were used for matching were also used as control variables for the initial model. The data collection period (which included a one-year pre-observation period and one year of follow-up) began in June 2017 and ended in March 2022. Accordingly, a part of the study period coincided with the COVID-19 pandemic. The related contact restrictions could have had an impact on both the personalized intervention and the general use of healthcare services [36,37]. To adjust for differences in healthcare use due to the pandemic, we included a control variable measuring the individual follow-up period under pandemic conditions in months per patient.

In addition to regression-based analyses of differences in direct costs between the control and intervention groups, cost-effectiveness was analyzed using incremental cost-effectiveness ratios (ICERs) [38] for the primary outcome (stroke recurrence). The incremental benefit (percentage change in the hazard rate of stroke recurrence during the follow-up period) was compared to the incremental costs of the intervention (difference between the direct costs covered by SHI, as calculated by the regression model, including the mean price of the intervention) [39,40]. The outcome measure thus reported the incremental costs per percentage change in the hazard of stroke recurrence. The calculation of the ICER was carried out exclusively on the basis of the model estimates, which control for several covariates. The ICER was determined for the total cohort, as well as for the subgroup of TIA patients.

In order to consider model and parameter uncertainty, and thus the uncertainty of the individual estimates from the regression models, a probabilistic sensitivity analysis was performed using bootstrapping [41]. For this purpose, 500 new samples were generated by drawing with replacement. The samples were drawn in such a way that the new intervention and control groups always had exactly the same weighted sample size as the original study groups. For each of these samples, two regression models were run: one for costs and one for recurrence rates. This resulted in 500 pairs of data points (incremental cost in euros versus effect as a percentage change in hazards). The results were presented in a cost-effectiveness plane. For this, the x-axis was scaled inversely, as negative percentage changes in hazards represented a positive effect of the intervention. Afterward, different willingness-to-pay scenarios per percentage change in hazards were considered and visualized. Based on the 500 bootstrapping results, a cost-effectiveness plane was estimated, which maps the probability that the intervention is cost-effective for various willingness-to-pay assumptions [33,42].

## 3. Results

### 3.1. Patient Characteristics

After matching, 1167 patients per group were included in the analyses. The mean age for both the control and the intervention group was 70 years. There was a slight predominance of male stroke patients in both groups (55% to 45%). The majority of patients suffered from an ischemic stroke (ICD-10 code I63: 82%), with TIA patients representing the next-largest group, at almost 15%. Hypertension (at 67%) was the most common pre-existing condition in both cohorts. The majority of patients were diagnosed using imaging techniques during the index stay, and only a small proportion initially required treatment in an intensive care unit using mechanical ventilation or a gastric tube. Details can be seen in [43].

### 3.2. Descriptive Analysis

Patients in the intervention group experienced fewer hospitalizations after their index stay and required fewer medical devices but recorded more outpatient physician consultations and more therapeutic services than those in the control group (see Table 4). An average of 9.83 intervention modules were billed by the stroke case managers.

The Wilcoxon rank sum test, applied due to the non-normal distribution of the cost data, indicated that total direct costs per patient post-stroke were significantly higher for the intervention group compared to the control group. Inpatient care constituted the largest cost component. Table 5 indicates that on average, the intervention group had lower costs related to inpatient care, pharmaceuticals, and medical devices but higher costs related to outpatient medical care, therapeutic services, and sick leave.

### 3.3. Regression Analysis

The log-linear regression model showed a significant difference in total direct costs between control and intervention groups: in the year following a stroke, the intervention group incurred 32.3% higher direct costs than the control group. Even after excluding intervention costs, direct expenses in the intervention group were still estimated to be 13.3% higher than those in the control group (see also Appendix A, Table A1).

### 3.4. Cost-Effectiveness Analysis

#### 3.4.1. Deterministic Analysis

For the total study population (all stroke patients), the direct costs were significantly higher in the intervention group than in the control group, with a difference of EUR 1384.78 in the regression analysis (β = 0.2802, exp(β) = 1.3234, 95% CI: [1.2384–1.4143], *p* < 0.0001). Additionally, a 5.32% increase in hazards was observed for the intervention group (HR = 1.0532, 95% CI: [0.7869–1.4096], *p* = 0.7274) [43]. This resulted in an incremental cost-effectiveness ratio (ICER) of EUR 260.30 per percentage point increase in hazards, indicating that the intervention was dominated (i.e., more costly and less effective). In the subgroup of TIA patients, the intervention group incurred costs that were EUR 231.17 higher than those of the control group (β = 0.2381, exp(β) = 1.2688, 95% CI: [1.0796–1.4912], *p* = 0.004), and the intervention had a non-significant protective effect in terms of the risk of recurrence (HR = 0.1563, 95% CI: [0.0216–1.1324], *p* = 0.0662). Therefore, with reference to the ICER for this subgroup, there was a EUR 2.74 per percentage point reduction in HR [43].

#### 3.4.2. Probabilistic Sensitivity Analysis

**Figure 1:**
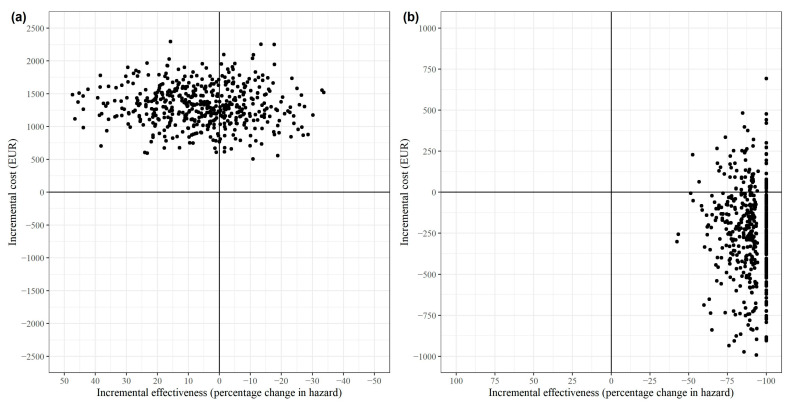
Cost-effectiveness plane for (**a**) all strokes and (**b**) the subgroup of TIA patients.

The bootstrapped cost-effectiveness estimates for both the total population (a) and the TIA subgroup (b) are displayed in the cost-effectiveness plane shown in Figure 1. The x-axis measured the incremental effect as a percentage reduction in hazards, consistent with the standard ICER interpretation that positive effects are preferable. In all 500 bootstrapped simulations for the total population, the case management intervention was more costly than the control. In 64% of these simulations, the intervention was less effective (north-west quadrant). In the remaining 36% of the simulations, the intervention was more effective (north-east quadrant). For the TIA subgroup, however, the intervention was more effective in all simulations, with 85% demonstrating dominance (more effective and less costly, south-east quadrant). In 15% of TIA subgroup simulations, the intervention was more costly (north-east quadrant). The large number of simulations with a 100% reduction in hazards for the TIA subgroup resulted from only one recurrence of stroke occurring in the intervention group. The exclusion of this patient during random sampling in bootstrapping occasionally led to this extreme effect.

**Figure 2:**
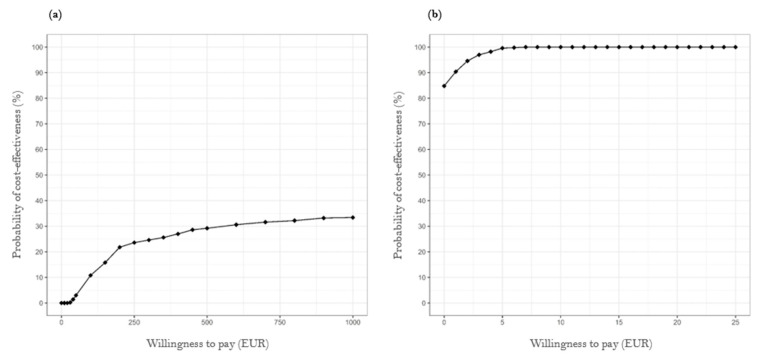
Cost-effectiveness acceptability curves of case management intervention for (**a**) all strokes and (**b**) the subgroup of TIA patients.

**Figure A1:**
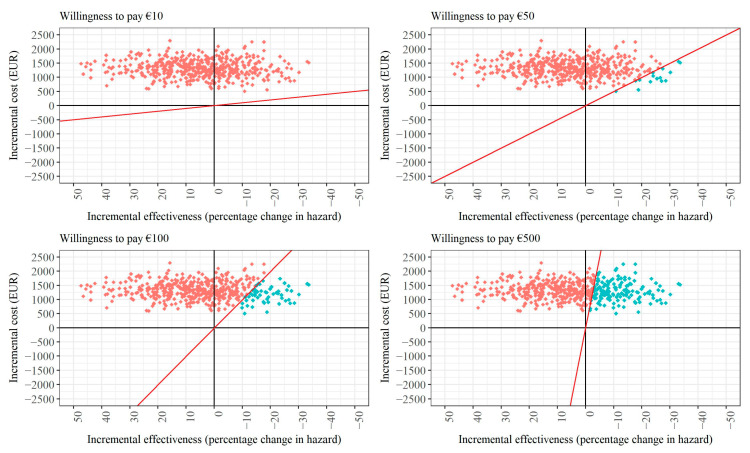
Cost-effectiveness planes for different willingness-to-pay (WTP) scenarios for case management after any type of stroke (assuming intervention costs must be covered). Note: The red line in each figure panel displays the WTP threshold. Red dots indicate draws that exceed the respective WTP threshold, while turquoise dots indicate draws that meet or fall below the WTP threshold.

**Figure A2:**
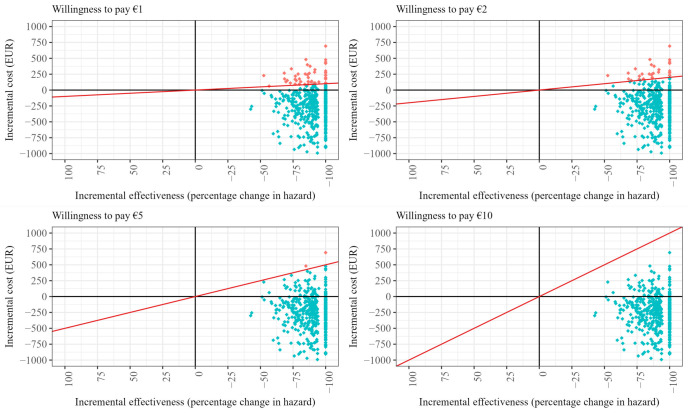
Cost-effectiveness planes for different willingness-to-pay (WTP) scenarios for case management after TIA (assuming intervention costs must be covered). Note: The red line in each figure panel displays the WTP threshold. Red dots indicate draws that exceed the respective WTP threshold, while turquoise dots indicate draws that meet or fall below the WTP threshold.

The cost-effectiveness acceptability curve illustrated in Figure 2a reveals that the intervention’s probability of cost-effectiveness for the total population remained low, even with a high willingness to pay, with a 1% probability at EUR 40. At a willingness to pay EUR 1000, the probability of the intervention being cost-effective was 33.4%. The curve flattened significantly beyond this point, indicating that an increase in the willingness to pay does not substantially enhance the probability of cost-effectiveness (see also Appendix B, Figure A1). This suggests that the intervention is unlikely to be considered cost-effective, even at higher willingness-to-pay levels. In contrast, the cost-effectiveness acceptability curve for TIA patients, presented in Figure 2b, shows that the probability of cost-effectiveness in this subgroup was at least 84.8% and reached 100% at a willingness to pay of just EUR 7 (see also Appendix C, Figure A2). 

## 4. Discussion

The introduction of case managers for first-ever stroke patients did not reduce the incidence of recurrent strokes and led to higher costs compared to the costs of standard care across the total stroke population in the first year after baseline assessment. In the TIA subgroup, however, the case management intervention had a non-significant protective effect, although the significance of this effect was limited by the small sample size. In both the total population and the TIA subgroup, the intervention group faced significantly higher total direct costs. Notably, the cost difference in the total group was significant, even after excluding intervention-specific costs, whereas in the TIA subgroup, it reached significance only when intervention costs were included.

The cost difference between the intervention and control groups was significant for only three non-intervention cost components (sick leave, therapeutic services, and outpatient care), with the intervention group incurring higher expenses in each component. Of these, sick leave contributed the largest absolute difference in costs. However, much of that difference was due to unit cost differences, which are not attributable to the intervention (i.e., higher incomes of intervention patients are independent of case management). Therapeutic services also led to a large absolute cost difference, driven by differences in use, which are attributable to the intervention. It can be assumed that the case managers will initiate appropriate healthcare and thus increase use. However, this increase in healthcare was not reflected in relevant improvements in outcomes. Finally, the difference in outpatient care costs, while statistically significant, led to only a small absolute cost difference. Furthermore, it should be noted that the log-linear regression model of costs did not control for the central study outcomes. Considering the fact that death leads to lower costs in the subsequent year, an influence on the level of costs incurred cannot be excluded. However, 36 patients died in the intervention group and 46 in the control group (3.1% vs. 3.9%; HR: 0.86; 95% CI: [0.58–1.28], *p* = 0.46). Hence, the mortality rate in both groups was low, and the difference observed was not significant, making an impact on the costs incurred rather unlikely [43].

Our deterministic and probabilistic health economic analyses demonstrate that the case management intervention is not cost-effective in the total stroke population, even for an arbitrarily high willingness to pay. This result aligns with the literature on case management approaches, which usually focus on severe cases [44]. In contrast, the STROKE OWL study excluded the most severe cases of stroke (mRS > 5) and included only a small number of severe cases (mRS = 4) [43]. This suggests that a case management intervention focused on more severe cases of stroke may be more cost-effective. In the TIA subgroup, however, we found that the intervention was cost-effective, even for a low willingness to pay. This is a surprising result since TIAs are the least severe form of stroke and are, therefore, usually not targeted by case management interventions [45].

These results should be interpreted in light of the limitations of our approach. First, as mentioned before, the total population in our study included only a small number of cases with severe stroke (mRS = 4), potentially leading to reduced effectiveness and cost-effectiveness estimates. Furthermore, the cost-effectiveness results in the total population were limited by the exclusion of several cost components, such as rehabilitation and nursing care, due to data heterogeneity. However, the costs for these excluded components were higher in the control group than in the intervention group; reliable data on these components may, therefore, have positively affected the cost-effectiveness of the intervention. In addition, only the number of hospital stays in the follow-up year was analyzed. An analysis of the length of subsequent hospital stays was not conducted. Accordingly, we cannot exclude the fact that there is any group difference. The TIA subgroup also suffered from a small sample size. In particular, only one stroke recurrence was recorded in the TIA intervention group, which led to some extreme effects in the probabilistic analysis.

The effectiveness of the intervention was assessed by comparing the hazards of stroke recurrence between intervention and control groups using hazard ratios (HRs). While HRs are clinically relevant, their application in cost-effectiveness calculations is challenging due to difficulties in interpretation and the relatively short duration they cover (i.e., one year). Consequently, the cost-effectiveness measures reported in this study are difficult for decision makers to use effectively in resource allocation decisions.

A more suitable long-term outcome measure would be the quality-adjusted life years (QALYs) accumulated until a patient’s death [46,47]. Although trial-based methods for collecting such long-term data are often impractical due to extended study durations (i.e., attrition and excessive costs), health economic models offer a robust alternative. These models can also integrate data from various studies, including utility measures not collected in the original study, thereby enhancing the relevance and comprehensiveness of the analysis [48,49]. As there is no defined WTP threshold in Germany, we referred to a hypothetical WTP. However, the uncertainty of making a wrong decision and the associated costs for decision makers are important. As there is no authoritative information on the risk preferences of German SHI decision makers, no further implications were considered.

Economic modeling provides more payer-relevant cost-effectiveness measures and also addresses a critical aspect of this study. Although the intervention showed a protective effect in the TIA group, this effect is not statistically significant. This observation suggests the potential value of a follow-up study. A modeling study can assist in planning such a study via a value of information analysis, specifically the expected value of sample information analysis [50]. This analysis quantifies the monetary value of reducing uncertainty through the collection of new evidence, helping to better inform resource allocation decisions and compare these benefits against the cost of collecting new evidence [51].

## 5. Conclusions

Our results show that the case management intervention is not cost-effective. Contrary to the initial hypothesis, the intervention is neither effective in terms of the recurrence rate nor less costly. The latter is not surprising, as the implementation of a new profession in the healthcare process can involve additional costs. However, these additional costs should at least lead to an improvement in care and thus to a reduction in the rates of recurrence and/or mortality in the long run. Contrary to the core concept of case management, the intervention does not target cost-intensive and complex individual cases.

Future studies should consider all relevant cost components and include a larger sample of patients with severe stroke (mRS > 3). Furthermore, our preliminary findings on cost-effectiveness in the TIA subgroup indicate a need for future studies with larger TIA samples, if indicated by the value of information analysis. In general, the design of case management interventions should align with the foundational ideas of the case management approach, although researchers should remain open to unexpected results in subgroups that are not usually targeted by such interventions (such as TIA patients).

Regardless of the specific target group, further health economic analyses should try to ensure maximum data homogeneity so that a comprehensive presentation of intervention effects on individual cost components, as well as on overall health costs, can be provided. Finally, the interpretability of results should be considered when designing health economic evaluation studies. The calculation of QALYs is recommended for a comprehensive estimation of cost-effectiveness.

## Figures and Tables

**Table 1:** Inclusion and exclusion criteria for study selection.

Criteria	Inclusion	Exclusion
CVD history	Initial stroke or TIA	Previous stroke
Age at study enrollment	≥18 years	<18 years
Initial mRS	0–4	>4
Long-term-care grade ^1^	0–3	>3
Previous living conditions		Previous residential care in a nursing home
Residence	Within the selected regions	Outside the selected regions
Insurance	Ongoing membership in one of the participating ^2^ SHI companies	Membership of another SHI or private health insurance
Serious comorbidities (ICD-10 code)		C00-C97 (malignant neoplasms) G30 (Alzheimer disease) G31 (other degenerative diseases of the nervous system, not elsewhere classified) F00-F09 (organic, including symptomatic, mental disorders) F10-F19 ^3^ (mental and behavioral disorders due to psychoactive substance use) F20-F29 (schizophrenia, schizotypal, and delusional disorders) F30 (manic episode) F31 (bipolar affective disorder)

^1^ Long-term care grade ranges from 1 to 5 and determines the need for care [31]; ^2^ Techniker Krankenkasse, IKK classic, BARMER, DAK, AOK Nordwest, and eight regional company health insurance providers; ^3^ excluding F17 (mental and behavioral disorders due to psychoactive substance use); CVD, cardiovascular disease; initial mRS, modified Rankin Scale determined during the initial hospital stay; SHI, statutory health insurance.

**Table 2:** Cost components measured.

Cost Component	Unit
Inpatient care	Number of hospital stays
Rehabilitation	Number of outpatient and/or inpatient rehabilitation stays
Outpatient medical care	Number of physician consultations
Pharmaceuticals	Number of daily defined doses
Therapeutic services and medical devices	Number of items
Sick leave	Number of sick leave days
Inpatient long-term care and home nursing	Number of prescribed services
Intervention costs	Average number of invoiced modules

**Table 3:** Modules invoiced and average intervention costs (*n* = 1105).

Modules	Services Provided per Patient ^1^ Mean (SD)	Costs per Service in EUR Mean (SD)
Basic service package (invoiced quarterly)	3.84 (0.59)	126.71 (19.38)
Enrollment and assessment	1 (0)	99.00 (0)
Rehabilitation visit	0.63 (0.48)	73.06 (55.71)
Reassessment/monitoring (face-to-face)	0.98 (0.60)	103.00 (62.72)
Additional contact (by phone)	2.30 (1.18)	113.87 (58.51)
Completion of contact	1 (0)	33.00 (33.00)
Determination of need for neuropsychological treatment	0.07 (0.25)	3.41 (12.53)
Full intervention		552.05 (117.78)

^1^ Minimum and maximum limits (if existing) have already been included in the calculations of the modules; use and cost calculations are based on the information provided by the participating SHI companies. *n*, number of observations; SD, standard deviation.

**Table 4:** Mean healthcare resource use per patient in the first year after a stroke.

Type of Healthcare	Control Group Mean (SD)	Intervention Group Mean (SD)
Inpatient care	1.01 (0.99)	0.88 (1.14)
Outpatient medical care	12.24 (12.59)	14.05 (6.44)
Pharmaceuticals	2348.27 (2469.47)	2434.22 (1243.01)
Therapeutic services	16.46 (31.65)	22.15 (41.65)
Medical devices	4.36 (6.84)	3.94 (6.45)
Sick leave	0.46 (1.61)	0.53 (2.20)
Intervention modules	n.a.	9.83 (1.81)
Rehabilitation ^1^	0.51 (0.58)	0.76 (0.66)
Residential nursing care ^1^	6.63 (8.04)	5.73 (10.88)
Home nursing care ^1^	2.86 (18.22)	1.93 (20.00)

^1^ These use components could not be included in the cost calculation due to persistent data heterogeneity caused by differences in statutory health-insurer-specific documentation. They have been included here for completeness, but comparability between the study groups is limited. n.a., not applicable; SD, standard deviation.

**Table 5:** Mean costs (EUR) per patient in the first year after a stroke.

Cost Components	Control Group Mean (SD)	Intervention Group Mean (SD)	SE	*p*-Value
Total direct costs	9554.56 (12,107.10)	10,600.62 (11,024.82)	405.27	<0.0001
Inpatient care	4351.08 (9100.98)	3929.56 (7759.13)	219.78	0.0511
Outpatient care	967.98 (1182.78)	1030.52 (1207.91)	42.87	0.0003
Pharmaceuticals	1231.56 (1907.69)	1210.93 (2285.85)	77.94	0.9948
Therapeutic services	779.75 (1482.07)	1153.47 (1741.06)	59.63	<0.0001
Medical devices	488.54 (1282.11)	442.85 (1049.18)	40.06	0.5715
Sick leave	1735.65 (5595.85)	2281.24 (6103.04)	212.83	<0.0001
Intervention	n.a.	552.05 (117.78)	n.a.	n.a.
Rehabilitation ^1^	2028.64 (4018.98)	2713.15 (3836.87)	138.92	<0.0001
Residential nursing care ^1^	3039.56 (45,157.71)	1826.04 (4069.23)	877.27	0.3208
Home nursing care ^1^	458.30 (3043.10)	313.13 (1277.40)	70.03	0.0874
Productivity losses	1389.07 (3776.01)	1741.09 (4244.00)	146.89	0.0548

^1^ These cost components could not be included in total costs used in the log-linear regression models due to persistent data heterogeneity caused by differences in statutory health-insurer-specific documentation. They have been included here for completeness, but comparability between the study groups is limited. n.a., not applicable; SD, standard deviation.

## Data Availability

The datasets generated and analyzed during this study are of particular sensibility and in high need for protection. Due to project-specific agreements of data protection, they are not publicly available. On reasonable request, possibilities of data access for external researchers have to be proved. Requests can be made to the corresponding author.

## Supplementary Materials

The following supporting information can be downloaded at https://www.mdpi.com/article/10.3390/healthcare12111157/s1, File S1. The Case Management-Intervention in STROKE OWL; Figure S1. Description of the Case Management process during the project STROKE OWL.

## Appendix A

**Table A1:** Results of the log-linear regression models for total direct costs (including intervention costs).

		Regression Coefficient (ß)	SE	95% CI		*p*-Value
				Lower	Upper	
All stroke patients ^2^	Intercept	8.3634	0.1117	8.1446	8.5822	<0.0001
	Age	−0.013	0.0015	−0.0159	−0.0101	<0.0001
	Type of stroke ^1^:					
	I60	−0.1702	0.574	−1.2952	0.9548	0.7668
	I61	0.2827	0.1114	0.0644	0.501	0.0112
	I63	0.4195	0.05	0.3215	0.5175	<0.0001
	Direct costs in the previous year	<0.001	<0.001	<0.001	<0.001	<0.0001
	Physician contacts in the previous year (outpatient)	0.0137	0.0017	0.0105	0.0169	<0.0001
	Diabetes mellitus	0.152	0.041	0.0716	0.2324	0.0002
	Index LOS	0.03	0.0025	0.025	0.035	<0.0001
	Intervention	0.2802	0.0339	0.2138	0.3466	<0.0001
Subgroup of TIA patients ^3^	Intercept	6.7568	0.2576	6.252	7.2617	<0.0001
	Age	0.0089	0.0037	0.0017	0.0162	0.0158
	Direct costs in the previous year	0.0001	<0.0001	0.0001	0.0001	<0.0001
	Hospital days in the previous year	−12.4399	3.9124	−20.1081	−4.7716	0.0015
	Physicians contacts in the previous year (outpatient)	0.0165	0.004	0.0087	0.0243	<0.0001
	Myocardial infarction	0.7811	0.3969	0.0031	1.5591	0.0495
	Diabetes mellitus	0.1896	0.1023	−0.011	0.3902	0.0644
	COPD	0.2156	0.1359	−0.0508	0.4819	0.1132
	Intervention	0.2381	0.0824	0.0766	0.3996	0.004

^1^ Reference category G45; ^2^ model quality AIC = 11,686.6428; R^2^ = 14.4%; ^3^ model quality AIC = 1929.9818; R^2^ = 19.34%; LOS, length of stay; COPD, chronic obstructive pulmonary disease.
