# Correction: Duevel et al. Secondary Prevention via Case Managers in Stroke Patients: A Cost-Effectiveness Analysis of Claims Data from German Statutory Health Insurance Providers. *Healthcare* 2024, *12*, 1157

**DOI:** 10.3390/healthcare12161609

**Published:** 2024-08-13

**Authors:** Juliane A. Duevel, Sebastian Gruhn, John Grosser, Svenja Elkenkamp, Wolfgang Greiner

**Affiliations:** AG 5—Health Economy and Healthcare Management, Faculty of Public Health, Bielefeld University, 33615 Bielefeld, Germany

**Error in Figure**

In the original publication [1], there was a mistake in Figure A2 as published. In the initial figure, the panel displaying results for a willingness-to-pay threshold of EUR 10 erroneously colored simulations below that threshold in red, suggesting they exceeded the threshold. The corrected Figure A2 appears below.

The authors state that the scientific conclusions are unaffected. This correction was approved by the Academic Editor. The original publication has also been updated.

## Figures and Tables

**Figure A2:**
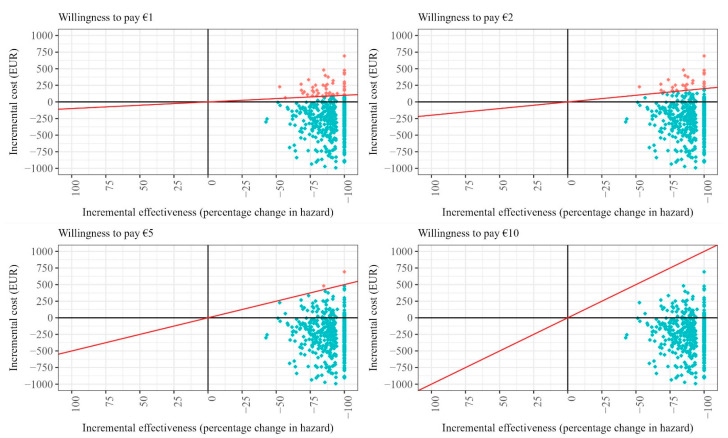
Cost-effectiveness planes for different willingness-to-pay (WTP) scenarios for case management after TIA (assuming intervention costs must be covered). Note: The red line in each figure panel displays the WTP threshold. Red dots indicate draws that exceed the respective WTP threshold, while turquoise dots indicate draws that meet or fall below the WTP threshold.
