# Supplementary material for: Secondary Prevention via Case Managers in Stroke Patients: A Cost-Effectiveness Analysis of Claims Data from German Statutory Health Insurance Providers
Source: Healthcare (Basel). 2024 Jun 6;12(11):1157. doi: 10.3390/healthcare12111157 (PMC11172283; doi:10.3390/healthcare12111157)
Supplement: Supplementary file 1 [file healthcare-12-01157-s001.zip › healthcare-3000532-supplementary.pdf]

**File S1. The Case Management-Intervention in STROKE OWL**

The case managers had existing professional qualifications from the fields of nursing, therapeutics or social work. In addition, they completed a case management training, which was carried out in accordance with the guidelines of the German Society for Care and Case Management [1] and were trained in stroke-specific topics, e.g. on neurological and neuropsychological basics, driving after stroke, physiotherapy/occupational therapy and medical devices as well as with regard to the implementation of the CM pathway, which was developed in the project (see Figure S.1).

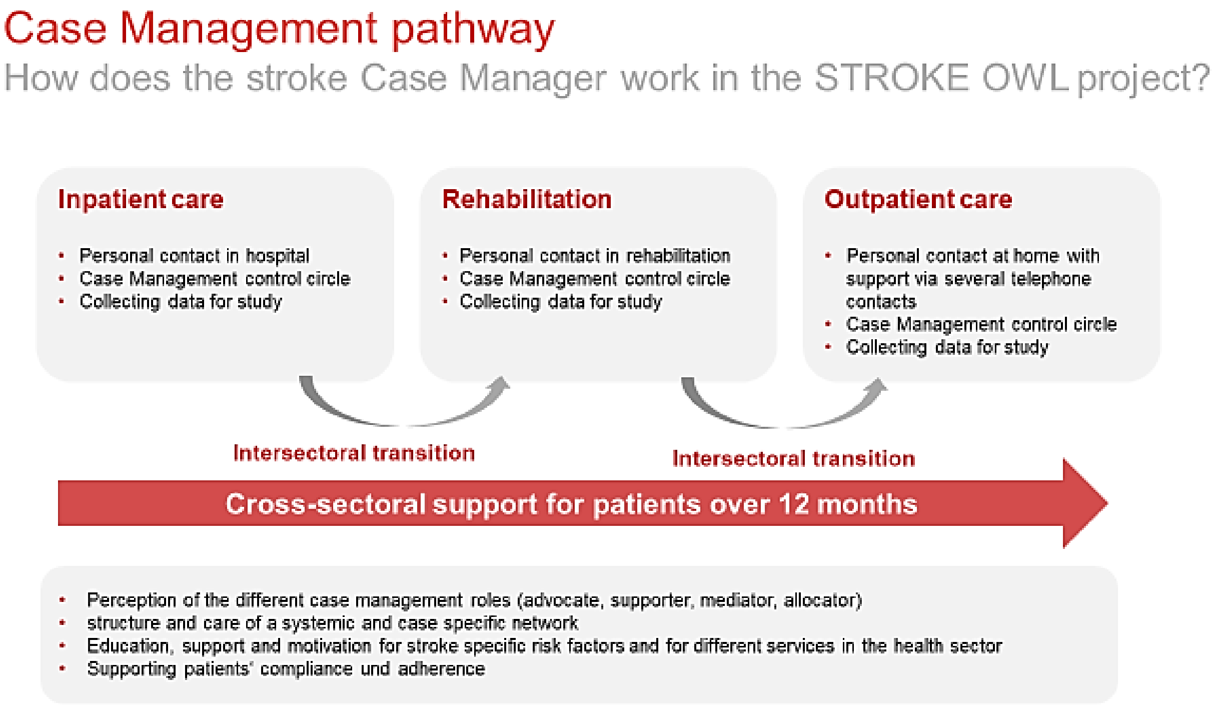


**Figure S1.** Description of the Case Management process during the project STROKE OWL [2]

The intervention started on the stroke unit in the hospital with the enrolment of the patient in the project. From the time of enrolment in the STROKE OWL project, the case manager supervised the patient and, if necessary, his or her relatives for one year. The case manager took on an advisory and coordinating function and was thus responsible for the coordination and continuity of health care from acute treatment to rehabilitation and outpatient health care. Due to the working method of a case manager, he/she did not take over the various medical responsibilities him/herself, but was in charge of an efficient organisation and coordination of the medical treatments, health care services and health professionals involved.

During the stroke unit stay an assessment including a risk factor plan and identified problems and resources of the patient and his situation were developed. This was used to determine the need for health care and assistance. The collection of the above-mentioned contents was standardized. If the patient received a rehabilitation programme, the assessment was revised during a visit to the rehabilitation clinic and, if necessary, the action plan was updated and revised. The re-assessment and the updating and revision of the action plan took place both during a

personal home visit shortly after discharge from inpatient treatment and within the framework of telephone contacts after six, nine and twelve months. In addition, the case manager had contact with the treating physicians (inpatient and outpatient) as well as with other health professionals involved.


**References**

1. Deutsche Gesellschaft für Care und Case Management. Standards und Richtlinien für die Weiterbildung Case Management im Sozial- und Gesundheitswesen und in der Beschäftigungsförderung 2019.
2. German Stroke Foundation. Report on the results STROKE OWL. 2023. https://innovationsfonds.g-ba.de/downloads/beschluss-dokumente/371/2023-02-23_STROKE%20OWL_Ergebnisbericht.pdf. Accessed 25 Apr 2023.
